# Course of Neuropsychiatric Symptoms during Flares of Systemic Lupus Erythematosus (SLE)

**DOI:** 10.1155/2017/2890436

**Published:** 2017-02-23

**Authors:** Gareth Garrett, Nicola Ambrose, Zaib Davids, Dorothea Bindman

**Affiliations:** University College London Hospitals NHS Trust, London, UK

## Abstract

We present the case of a seventeen-year-old girl who presents with an interesting course of neuropsychiatric symptoms during several flares of SLE. The patient was diagnosed at the age of thirteen and has had four flares in total. The latter two flares included cutaneous and neuropsychiatric symptoms. The most recent flare occurred when she was aged seventeen. She had cutaneous symptoms which coincided with an episode of hypomania. Her mental state further deteriorated following steroid treatment. She exhibited affective and psychotic symptoms. Treatment with cyclophosphamide and olanzapine was associated with an improvement in both cutaneous and neuropsychiatric symptoms. Previously aged sixteen the patient had presented with cutaneous symptoms and a moderate depressive episode which was also exacerbated by steroid treatment. The patient's mood improved when the dose of oral steroids was reduced to a daily dose of 15–20 mg prednisolone.

## 1. Introduction

This case highlighted potential challenges in the treatment of cerebral lupus given the complexity in making a clinical diagnosis and the potential for neuropsychiatric side effects associated with treatment.

## 2. Case Presentation

A 17-year-old girl with a history of SLE who had received treatment with regular azathioprine for at least one year presented to rheumatology clinic with a one-week history of a rash. On examination the patient had a malar rash and oral mucosal ulceration. Neurological examination was unremarkable. The patient reported a recent history of hypomanic symptoms that had ceased without treatment immediately prior to the onset of the rash.

She reported a week-long history of poor sleep, racing thoughts, irritability, and grandiose overvalued ideas. She had held the belief that she was destined to open a French language school and to start a clothing line, both of which were certain to be a worldwide success. When challenged on these ideas by her mother she would become markedly irritable. She had spent time through the nights making collages and mood boards for the fashion line. Her mother confirmed that these ideas were totally out of character and far beyond her capabilities despite her interest in the fashion industry. She had become irritable with several friends both in person and on social media. The onset and termination of these symptoms was acute, though she remained mildly irritable.

The patient had had three previous flares. The first flare occurred when the patient was thirteen. The first two flares had cutaneous and renal involvement but no psychiatric symptoms. The third flare had occurred one year previously when she presented with a malar rash and an acute depressive episode of moderate severity, which was exacerbated by treatment with pulsed steroids. During this episode the patient complained of low mood and low energy and had thoughts to jump out a high storey window of the hospital. These thoughts were not acted upon. This depressive episode gradually resolved once the oral steroid dose was reduced to 15–20 mg prednisolone daily.

There was no family history of mental illness.

Bloods tests revealed a raised DsDNA and low C3. Lumbar puncture showed normal protein, white cell count, and microscopy. CSF oligoclonal bands were negative.

MRI brain was reported as follows.

“The intracranial appearances are normal. A few punctate foci of contrast uptake, most conspicuous within the posterior fossa, appear related to normal vessels. There is no convincing pathological parenchymal or meningeal enhancement.”

Differential diagnosis included SLE, a viral or bacterial encephalitis including the possibility of an atypical infection given the patient's immunosuppression, a primary psychiatric disorder, normal adolescent behaviour, adverse effects of medications, illicit drug use, and lymphoma.

The diagnosis of a lupus flare with cutaneous and neuropsychiatric involvement was made and steroid treatment was initiated.

Several hours following the first dose of methylprednisolone, the patient's mental state deteriorated. She experienced sleep disturbance, disorientation, and agitation and expressed paranoid delusions. She held the belief that the family were being monitored by an unknown agency who were convinced her parents had stolen medical devices from the ward. She was suspicious and hypervigilant around staff whom she believed were constantly talking about her. She denied that she was unwell and made attempts to leave the ward against advice complaining that she needed to get back to school or attend the gym. There was no perceptual abnormality and she was fully orientated.

The patient was initially commenced on a three-day course of intravenous methylprednisolone 250 mg, followed by a switch to oral prednisolone which was gradually reduced and stopped over six weeks. She received six infusions every four weeks of cyclophosphamide 750 mg, the first of which was given during the admission described.

Her cutaneous symptoms improved rapidly. Following the pulsed steroid treatment and subsequent deterioration in her mental state, there was a period of watchful waiting for several days, followed by treatment with olanzapine titrated up to a dose of 7.5 mg once daily. This resulted in an improvement in her psychotic symptoms. The patient was discharged from hospital.

The patient's mental state was followed up closely over the following month and olanzapine gradually reduced and discontinued. At three-month follow up the patient's cutaneous and psychiatric symptoms remained in remission.

## 3. Discussion

Neuropsychiatric manifestations in SLE are common [1]. At University College Hospital London one in four patients have neuropsychiatric involvement. A recent review suggested that neuropsychiatric symptoms were more common in juvenile-onset SLE [2]. Nineteen neuropsychiatric syndromes in SLE have been recognised by the American College of Rheumatology [3]. These cover a range of presentations including headache, seizure disorders, and cerebrovascular disease. Mood disorder and psychosis are included as separate syndromes [3].

This case demonstrates the potential for affective disturbance including both depressive and manic episodes as well as the potential for psychotic symptoms. The pathogenesis of such conditions is not fully understood. Several potential mechanisms have been identified, including antibody-mediated neurotoxicity, vasculopathy due to anti-phospholipid antibodies, and cytokine-induced neurotoxicity [4].

The course of our patient's symptoms was of particular interest. During the most recent flare, there appeared to be a temporal relationship between cutaneous and hypomanic symptoms. It is curious, however, that the hypomanic symptoms appeared to have remitted before the rash prompted a rheumatology assessment. It is therefore a point for debate whether one should regard that episode as evidence for a functional psychiatric disorder, though the temporal relationship between depressive and cutaneous symptoms during a previous flare could be used for evidence against this. It would certainly be our view that this was a manifestation of cerebral lupus.

An interesting aspect to this case was that steroid treatment appeared to exacerbate both depressive and manic symptoms during two independent flares. Of course the potential for such side effects is well recognised [5], as well as some evidence that other immunosuppressants can exacerbate episodes of functional mental illness [6]. In addition, it is well known that individuals with chronic diseases are at greater risk of developing affective disorders [7].

The case demonstrates the potential difficulties in the assessment and treatment of patients presenting with neuropsychiatric symptoms of SLE, especially those likely to experience psychiatric side effects from therapy. In such patients there may be a degree of uncertainty in regard to the aetiology of the disturbance, especially should there be no other organ system involvement. The case demonstrates the potential benefits from joint care between rheumatology and psychiatry, though this is not always available in the inpatient setting.

Our patient was judged to require treatment with an oral antipsychotic agent due to her behaviour impacting on her medical treatment and the intensity of her psychotic symptoms. Olanzapine was selected for its sedative properties. There was an effort to discontinue the antipsychotic promptly given the potential for metabolic side effects and undue sedation once the patient had recovered. The evidence for the choice of antipsychotic agent in cerebral lupus is limited. Clinicians may be led by the side effect profile, tolerability, and patient choice of antipsychotic agent.

It is likely that both inflammatory and iatrogenic factors contributed to the patient's presentation, which would prompt careful consideration in the treatment of subsequent flares.
